# Exploratory analysis of the French version of the beliefs about medicines questionnaire in patients with severe mental disorders: Factorial structure and reliability in specific populations of schizophrenic, bipolar and depressive patients

**DOI:** 10.1371/journal.pone.0173267

**Published:** 2017-03-03

**Authors:** Ludovic Samalin, Ingrid de Chazeron, Raoul Belzeaux, Pierre-Michel Llorca

**Affiliations:** 1 Department of Adult Psychiatry, CHU Clermont-Ferrand, University of Auvergne, EA, Clermont-Ferrand, France; 2 Bipolar Disorder Unit, Institute of Neuroscience, Hospital Clinic, University of Barcelona, IDIBAPS, CIBERSAM, Barcelona, Catalonia, Spain; 3 Fondation FondaMental, Créteil, France; 4 Bipolar Disorder Expert Centre, Hôpital Sainte Marguerite, APHM, Marseille, France; 5 Department of Psychiatry, McGill Group for Suicide Studies, Douglas Mental Health University Institute, McGill University, Montreal, Quebec, Canada; Universite de Bretagne Occidentale, FRANCE

## Abstract

**Objectives:**

The aims of our study were to explore the factor structure and psychometric properties of the French version of the Beliefs about Medicines Questionnaire (BMQ) in patients with severe mental illness and in specific populations of patients with schizophrenia, bipolar disorder and major depressive disorder.

**Methods:**

A cross-sectional study including patients with schizophrenia, bipolar disorder and major depressive disorder was conducted (n = 150). Principal component analysis (PCA), reliability and validity of the French version of the BMQ were performed.

**Results:**

PCA revealed a two-factor structure similar to the original structure for the BMQ-Specific scale but only a one-component solution for the BMQ-General scale in both the total sample and the three subgroups. These subscales have satisfactory internal consistency. Validity was supported by the significant correlations of all BMQ subscales with the Drug Attitude Inventory.

**Conclusion:**

The French version of the BMQ appears as a three-dimensional scale and presents satisfactory psychometric properties for use in patients with severe mental illness as well as specific populations of patients with schizophrenia, bipolar disorder and major depressive disorder.

## Introduction

Psychotropic medications are the cornerstone and the standard of care for the treatment of severe mental illness (SMI). However, medication non-adherence is frequent in these chronic illnesses with an estimated prevalence rate in schizophrenia of about 50% [1] and in mood disorders of about 40% [2]. Non-adherence is associated with poor clinical outcomes in these patients such as higher risk of recurrence/relapse and hospitalization [3, 4], poorer psychosocial functioning [5] and higher rates of suicide [4, 6]. The negative attitudes and beliefs toward medication as well as poor insight, poor therapeutic alliance and low social support represent the most important factors associated with non-adherence [2, 7, 8].

To improve the understanding of non-adherence to medication, Horne et al. developed the Beliefs about Medicines Questionnaire (BMQ) on the basis of social cognitive models, such as the extension of Leventhal’s self-regulation model [9]. This is the most specific model about medication, suggesting that adherence is heavily influenced by the patients’ own beliefs and representation of the illness. This scale measures the specific patient’s perception of the need to take medication and concerns about it (BMQ-Specific, with two subscales named “Necessity” and “Concern”) as well as the general beliefs about pharmacotherapy (BMQ-General, with two subscales named “Harm” and “Overuse”).

This scale has been used in relation to a broad range of chronic diseases in different language versions [10]. Studies in SMI patients using the BMQ have shown its potential to explore and understand the determinants of non-adherence and to help the clinician to determine individual targets for interventions to improve patients’ negative beliefs. In these studies with schizophrenic patients [11], bipolar patients [12] or patients with major depressive disorder [13], higher adherence was associated with stronger perceived necessity of psychotropic treatment and fewer concerns regarding treatment. These specific beliefs (BMQ-Specific) about medication impacted medication adherence independently of the general beliefs (BMQ-General) toward pharmacotherapy [11].

In France, one study has validated the BMQ in patients with diabetes and HIV and found the same factorial structure as the English original version [14] even if the process does not follow steps recommended for validation scale in the COSMIN guidelines [15]. Theoretical concepts need to be explored in order to confirm that the French version of this scale is a suitable instrument in SMI patients. Most validation studies of BMQ in SMI patients were conducted in a mixed sample of psychiatric patients despite their clinical heterogeneity [9, 16–18]. Specific studies in defined samples of schizophrenic, bipolar and depressive patients are needed in order to support the stability of the factor structure and the psychometric properties of the BMQ among them.

The aims of our study were to explore the factor structure and some psychometric properties of the French version of the BMQ in SMI patients and to describe the factor structure and psychometric properties in specific populations of patients with schizophrenia, bipolar disorder and major depressive disorder.

## Materials and methods

### Participants

All adult patients (outpatients or inpatients) treated by at least one psychotropic medication (i.e. antipsychotic, mood stabilizer or antidepressant), who met the diagnosis of schizophrenia, bipolar disorder or major depressive disorder according to DSM-IV-TR criteria were recruited consecutively at two different French mental health hospital sites during a 1-year period. Exclusion criteria were inability to understand the self-administered questionnaires or the interviewer because of language problems or a learning disability. After the procedure was fully explained, written informed consent was obtained from all participants. Formal assessment of capacity was performed where applicable; patients lacking the capacity to provide written, informed consent were not approached to participate. The study was approved by the Institutional Review Board (CECIC Rhône-Alpes-Auvergne, Grenoble, IRB 5921) and was conducted in accordance with the Helsinki Declaration of 1975, as revised in 1983.

### Measures

Socio-demographic and clinical data were collected through a patient interview. The attitudes toward medication were assessed using the French versions of the BMQ and the 10-item Drug Attitudes Inventory (DAI). All patients filled in the BMQ and the 10-item DAI simultaneously.

The BMQ is an 18-item self-questionnaire assessing patients’ beliefs about their medication [9]. The original version comprises a Specific and a General scale, and each has two subscales. The BMQ-Specific has a 5-item Necessity scale that assesses beliefs about the necessity of the prescribed medication and a 5-item Concerns scale that assesses concerns about potential adverse effects of treatment. The BMQ-General assesses more general beliefs or social representations about drugs, including 8 items in two subscales of 4-item (Harm and Overuse subscales). Each item of the questionnaire is rated on a 5-point Likert scale (1 = “strongly disagree” to 5 = “strongly agree”). Each Necessity and Concerns scale ranges from 5 to 25 and each Harm and Overuse scale ranges from 4 to 20. Higher Necessity scores represent stronger perceptions (or positive beliefs) of personal need for the medication, and higher Concerns scores represent stronger concerns (or negative beliefs) about the potential negative effects of the medication. Higher scores on the General subscales represent more negative views about medicines.

In this study, the French version of the BMQ was used [14]. It showed satisfactory properties with the same factorial structure as the original version (two factors for the BMQ-Specific scale and two factors for the BMQ-General scale) in both diabetes and HIV patients.

The 10-item DAI is a self-report instrument that includes 10 questions on the subjective response to medication regarding both positive and negative effects [19, 20]. The respondents rate statements as true or false. The total score ranges from -10 to +10, a positive total score indicating a positive attitude towards psychiatric medication and a negative total score indicating a negative attitude towards psychiatric medication. The French version of the DAI showed satisfactory psychometric properties similar to the original version in SMI patients [21]. Despite some limitations (e.g. dichotomous ratings, items about both subjective states and attitudes towards medication), the 10-item DAI was used for the criterion validity of the BMQ because it is one of the most frequently used patient self-report measures to assess attitudes towards medication and can also predict medication adherence in SMI patients [22].

### Statistical analysis

Exploratory factor analysis was performed to assess measurement items within scale to check dimensionality, factor structure, and strength of factor loadings. Construct validity was first explored using principal component analysis (PCA) with varimax rotation for all included patients (considered as SMI patients). Then, PCAs were conducted for the three subgroups of patients (schizophrenia, bipolar disorder and major depressive disorder). A PCA was performed for each scale (Specific and General). In line with recommendations [23, 24], factors generated by the PCA were extracted as valid if at least two of the following criteria were met: i) eigenvalues were equal to or greater than unity [25]; ii) scree (Cattel’s) test (by the identification of the point of inflection in the scree plot) [26] and; iii) eigenvalues exceed the 95^th^ percentile of the corresponding simulated eigenvalue from Horn’s parallel analysis [27].

Mean, standard deviation and median were calculated for the BMQ subscales. Normality was explored based on skewness and kurtosis values. Floor and ceiling effects were measured by the percentage of patients with minimum and maximum scores respectively and should be below 15% to meet the acceptable measurement [28].

Cronbach’s alpha was used to estimate internal consistency. Pearson’s correlation coefficient was used for inter-factor correlation and factor correlation with DAI in order to establish criterion validity.

All statistical tests were two-tailed and the significance level was set at 5%. Statistical analyses were performed using SAS 9.3 ® software and Monte-Carlo parallel analysis software.

## Results

A total of 150 treated patients with schizophrenia (n = 50), bipolar disorder (n = 50) and major depressive disorder (n = 50) were recruited (Table 1). The mean ± SD age was 51.3 ± 16.6 and more than half of the patients were males (67%) without employment (74.7%). The mean ± SD illness duration was 14.3 years ± 10.4. Second-generation antipsychotics were the most frequent medication used in schizophrenic patients (98.0%), antidepressants the most frequent medication used in major depressive disorder (92.0%) and mood stabilizers the most frequent treatment prescribed for bipolar patients (86.0%).

**Table 1:** Socio-demographic and clinical characteristics of the study sample.

		Schizophrenia (n = 50)	Bipolar disorder (n = 50)	Major depressive disorder (n = 50)	Total (n = 150)
Age (years, SD)		40.9 (11.2)	54.9 (16.7)	58.3 (16.1)	51.3 (16.6)
Gender (n, % males)		34 (68.0)	17 (34.0)	16 (32.0)	67 (44.7)
Marital status, single (n, %)		41 (82.0)	25 (50.0)	32 (66.7)	98 (66.2)
Employment (n,%)		5 (10.0)	15 (30.0)	18 (36.0)	38 (25.3)
Educational level (n,%)	Primary	31 (62.0)	17 (34.0)	21 (43.8)	69 (46.6)
	Secondary	12 (24.0)	14 (28.0)	16 (33.3)	42 (28.4)
	Higher	7 (14.0)	19 (38.0)	11 (22.9)	37 (25.0)
Illness duration (years, SD)		13.9 (8.3)	16.2 (10.1)	13.0 (12.2)	14.3 (10.4)
CGI-S (score, SD)		4.6 (0.9)	3.4 (1.1)	3.9 (0.9)	3.9 (1.1)
Medications (n,%)	FGA	14 (28.0)	4 (8.0)	5 (10.0)	23 (15.3)
	SGA	49 (98.0)	21 (42.0)	10 (20.0)	80 (53.3)
	Mood stabilizer	15 (30.0)	43 (86.0)	20 (40.0)	78 (52.0)
	Antidepressant	21 (42.0)	21 (42.0)	46 (92.0)	88 (58.7)
	Benzodiazepine	21 (42.0)	19 (38.0)	24 (48.0)	64 (42.7)

CGI-S, Clinical Global Impression–Severity scale; FGA, first-generation antipsychotic; SGA, second-generation antipsychotic.

### Principal component analysis

**Fig 1:**
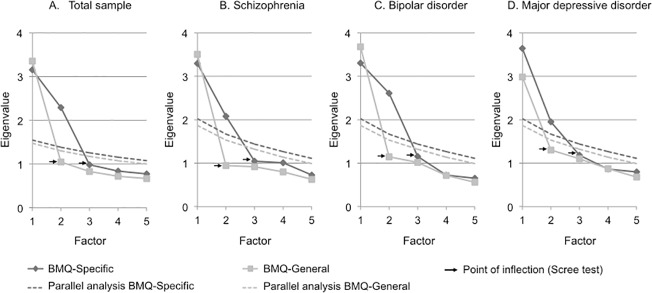
Scree plots for the PCAs of the BMQ scales in total sample and specific populations. Scree plots for the PCAs of the BMQ scales in total sample (A); patients with schizophrenia (B); patients with bipolar disorder (C); and patients with major depressive disorder (D). BMQ, Beliefs about Medicines Questionnaire.

PCA of the BMQ-Specific revealed a two-component solution for the total sample and the three subgroups of patients that clearly replicated the original structure (Fig 1, Table 2).

**Table 2:** Number of factors to extract according to different criteria.

		Kaiser	Scree test	Parallel analysis	Factor decision to extract
Total sample (n = 150)	BMQ-Specific	2	2	2	2
	BMQ-General	2	1	1	1
Schizophrenia (n = 50)	BMQ-Specific	4	2	2	2
	BMQ-General	1	1	1	1
Bipolar disorder (n = 50)	BMQ-Specific	3	2	2	2
	BMQ-General	3	1	1	1
Major depressive disorder (n = 50)	BMQ-Specific	3	2	2	2
	BMQ-General	3	1	1	1

BMQ, Beliefs about Medicines Questionnaire.

Kaiser recommends extracting factors with eigenvalue ≥ 1; Scree (Cattel’s) test recommends extracting factors above the elbow of the scree plot; Horn’s parallel analysis recommends extracting factors above the randomly generated eigenvalue (95th percentile).

In the total sample, the first factor, “Necessity” in the original scale, explained 32% of the variance and the second, “Concerns” in the original scale, explained 23% (Table 3). All items loaded higher than 0.40 on the respective factors. No item loaded high on two factors. In the subgroups of patients with schizophrenia, bipolar disorder and major depressive disorder, the first factor (Necessity) explained 33%, 33% and 36% of the variance respectively, and the second factor (Concerns) explained 21%, 26% and 20% respectively (Table 4). All items loaded higher than 0.40 on the respective factors, except for item 5 (“I sometimes worry about the long-term effects of my medicines”) in the schizophrenia group, which obtained small loading on both factors, and item 9 (“I sometimes worry about becoming too dependent on my medicines”) in the major depressive disorder group, which loaded higher on the Necessity factor.

**Table 3:** Principal component analysis of the BMQ scales for patients with severe mental disorders (n = 150).

	Factor 1	Factor 2	Factor 1
	Necessity	Concerns	General
BMQ-Specific items			
Item 1 : My health, at present, depends on my medicines	**0.86**	-0.02	
Item 3 : My life would be impossible without my medicines	**0.77**	0.09	
Item 4 : Without my medicines I would be very ill	**0.83**	0.12	
Item 7 : My health in the future will depend on my medicines	**0.82**	-0.09	
Item 10 : My medicines protect me from becoming worse	**0.57**	-0.33	
Item 2 : Having to take medicines worries me	-0.05	**0.82**	
Item 5 : I sometimes worry about the long-term effects of my medicines	0.14	**0.57**	
Item 6 : My medicines are a mystery to me	-0.16	**0.52**	
Item 8 : My medicines disrupt my life	-0.20	**0.67**	
Item 9 : I sometimes worry about becoming too dependent on my medicines	0.18	**0.68**	
BMQ-General items			
Item 11 : Doctors use too many medicines			**0.67**
Item 12: People who take medicines should stop their treatment for a while every now and again			**0.59**
Item 13: Most medicines are addictive			**0.49**
Item 14: Natural remedies are safer than medicines			**0.73**
Item 15: Medicines do more harm than good			**0.64**
Item 16: All medicines are poisons			**0.60**
Item 17: Doctors place too much trust in medicines			**0.78**
Item 18: If doctors had more time with patients they would prescribe fewer medicines			**0.63**
% variance explained after extraction	31.59	22.88	41.91

BMQ, Beliefs about Medicines Questionnaire. Factor loadings > 0.40 of the PCA are shown in bold.

**Table 4:** Principal component analysis of the BMQ scales for patients with schizophrenia, bipolar disorder and major depressive disorder.

	Schizophrenia (n = 50)			Bipolar disorder (n = 50)			Major depressive disorder (n = 50)		
	Factor 1	Factor 2	Factor 1	Factor 1	Factor 2	Factor 1	Factor 1	Factor 2	Factor 1
	Necessity	Concerns	General	Necessity	Concerns	General	Necessity	Concerns	General
BMQ-Specific items									
Item 1	**0.82**	-0.12		**0.89**	0.06		**0.86**	-0.09	
Item 3	**0.83**	0.10		**0.71**	0.05		**0.77**	-0.01	
Item 4	**0.82**	0.23		**0.84**	0.04		**0.83**	0.02	
Item 7	**0.69**	-0.39		**0.87**	-0.17		**0.82**	0.08	
Item 10	**0.57**	-0.38		**0.55**	-0.29		**0.60**	-0.37	
Item 2	-0.22	**0.80**		-0.06	**0.84**		0.28	**0.78**	
Item 5	0.06	0.19		0.21	**0.70**		0.17	**0.68**	
Item 6	-0.15	**0.43**		-0.13	**0.57**		-0.09	**0.56**	
Item 8	-0.16	**0.75**		-0.11	**0.75**		-0.24	**0.62**	
Item 9	0.14	**0.79**		-0.06	**0.75**		**0.64**	0.21	
BMQ-General items									
Item 11			**0.58**			**0.79**			**0.66**
Item 12			**0.75**			**0.56**			**0.43**
Item 13			**0.52**			**0.42**			**0.54**
Item 14			**0.79**			**0.73**			**0.60**
Item 15			**0.48**			**0.79**			**0.63**
Item 16			**0.72**			**0.60**			**0.48**
Item 17			**0.78**			**0.83**			**0.77**
Item 18			**0.59**			**0.61**			**0.71**
% variance explained	33.03	20.76	43.82	33.11	26.13	46.04	36.44	19.50	37.33

BMQ, Beliefs about Medicines Questionnaire. Factor loadings > 0.40 of the PCA are shown in bold.

PCA of the BMQ-General revealed a one-component solution for the total sample and the three subgroups of patients (Fig 1, Table 2). This factor explained 42% of the variance in the total sample and 44%, 46% and 37% of the variance in the schizophrenia group, bipolar disorder group and major depressive disorder group respectively. In the total sample and the three subgroups, this factor comprised all items of the BMQ-General scale with all items loaded higher than 0.40. It indicated that SMI patients, as well as patients with schizophrenia, bipolar disorder and major depressive disorder, do not distinguish the perceived Harm and Overuse of medication as the original scale.

### Descriptive statistics

The mean scores of the BMQ-Specific subscales and the BMQ-General scale (Necessity = 19.04 ± 4.27, Concerns = 15.08 ± 4.37, General = 21.50 ± 5.89) in the total sample (Table 5) were similar to the previous studies in SMI patients [17]. The mean scores of the three factors were also similar in the three subgroups of patients without significant difference between groups. There were no significant floor or ceiling effects for subscales in different psychiatric groups (<15%) and results also revealed no severe normality violations (skewness and kurtosis) (Table 5).

**Table 5:** Mean scores, floor effect, ceiling effect, skewness and kurtosis of the BMQ subscales in the total sample and subgroups of patients.

		Raw mean (SD)	Raw median (IQR)	Min	Max	Floor (%)	Ceiling (%)	Skewness	Kurtosis
Total sample	Necessity	19.04 (4.27)	20.0 (17–22)	6	25	0.67	10.00	-0.69	0.19
	Concerns	15.08 (4.37)	15.0 (12–18)	5	25	0.67	0.67	-0.05	-0.72
	General	21.50 (5.89)	22.0 (17–26)	8	37	2.00	0.67	-0.09	-0.23
Schizophrenia	Necessity	19.50 (4.02)	20.0 (17–22)	10	25	2.00	12.00	-0.60	-0.39
	Concerns	14.56 (3.99)	15.0 (12–18)	7	21	2.00	6.00	-0.21	-0.97
	General	20.98 (6.36)	20.5 (16–25)	8	37	2.00	2.00	0.41	0.04
Bipolar disorder	Necessity	19.36 (4.40)	19.5 (17–23)	6	25	2.00	12.00	-0.83	0.64
	Concerns	15.64 (5.13)	15.5 (11–20)	5	25	2.00	2.00	-0.09	-1.02
	General	21.56 (6.26)	23.0 (17–26)	8	33	2.00	4.00	-0.33	-0.74
Major depressive disorder	Necessity	18.26 (4.35)	19.0 (16–21)	7	25	2.00	6.00	-0.66	0.35
	Concerns	15.04 (3.91)	16.0 (12–18)	7	24	2.00	2.00	-0.07	-0.33
	General	21.96 (5.04)	23.0 (19–25)	8	35	2.00	2.00	-0.49	0.67

Concerns, Beliefs about Medicines Questionnaire (BMQ) Specific-Concerns; General, BMQ General; IQR, interquartile range; Max, maximum; Min, minimum; NA, not applicable; Necessity, BMQ Specific-Necessity; SD, standardized deviation.

### Reliability

There was good or acceptable consistency between items in the total sample, Cronbach’s alpha for the BMQ-Specific Necessity and Concerns and the BMQ-General were 0.83, 0.68 and 0.80 respectively (Table 6). There was good consistency between items in the three subgroups except for the BMQ-Specific Concerns in patients with schizophrenia and major depressive disorder, which showed questionable values (Table 6). Inter-factor correlations were acceptable with weak to moderate relationships in both the total sample and the three subgroups of patients (Table 6). No significant relationships were found between the BMQ-Specific subscales for all groups.

**Table 6:** Internal consistency, inter-item correlations, inter-factor correlation and factor correlation with the DAI scale.

		Cronbach’s alpha (original study [9])	Inter-item correlation	Inter-factor correlation		Convergent validity with DAI
				Concerns	General	
Total sample	Necessity	0.83 (0.55–0.86)	0.217–0.724	-0.082	0.354***	0.453***
	Concerns	0.68 (0.63–0.80)	0.126–0.467		0.538***	-0.459***
	General	0.80 (NA)	0.214–0.541			-0.503***
Schizophrenia	Necessity	0.81 (0.55–0.86)	0.248–0.690	-0.221	-0.451**	0.551***
	Concerns	0.62 (0.63–0.80)	0.042–0.552		0.627***	-0.569***
	General	0.81 (NA)	0.166–0.704			-0.619***
Bipolar disorder	Necessity	0.84 (0.55–0.86)	0.151–0.745	-0.114	-0.391**	0.533***
	Concerns	0.78 (0.63–0.80)	0.177–0596		0.514 ***	-0.382**
	General	0.82 (NA)	0.083–0.692			-0.446**
Major depressive disorder	Necessity	0.85 (0.55–0.86)	0.229–0.738	0.137	-0.194	0.397**
	Concerns	0.56 (0.63–0.80)	0.064–0.522		0.413**	-0.436**
	General	0.75 (NA)	0.066–0.522			-0.476***

DAI, Drug Attitude Inventory scale; Concerns, Beliefs about Medicines Questionnaire (BMQ) Specific-Concerns; General, BMQ General; Necessity, BMQ Specific-Necessity

** p < 0.01.

*** p < 0.001.

Higher Necessity scores represent stronger perceptions (or positive beliefs) of personal need for the medication. Higher Concerns scores represent stronger concerns (or negative beliefs) about the potential negative effects of the medication. Higher scores on the General subscales represent more negative views about medicines. Positive DAI score indicates positive attitude towards medication.

### Validity

The BMQ-Specific subscales and the BMQ-General scores significantly correlated with the DAI scores in the total sample and the three subgroups of patients, indicating that the BMQ assesses a construct that is similar to the DAI in patients with SMI as well as patients with schizophrenia, bipolar disorder and major depressive disorder (Table 6).

## Discussion

The French version of the BMQ partially supports the original factor structure (BMQ-Specific, two factors; BMQ-General, two factors) in SMI patients. Our findings replicate the original factor structure of the BMQ-Specific scale but not the BMQ-General, for which a monofactorial solution was found. Similarly, factor analysis in the specific populations of patients revealed the same factor structure as the total sample. The French version of the BMQ presented satisfactory psychometric properties for use in SMI patients. It is also psychometrically sound for measuring the beliefs about medications in patients with schizophrenia, bipolar disorder and major depressive disorder, despite their clinical heterogeneity.

In contrast with the French version validated in patients with diabetes and HIV [14] and similar to previous validation in schizophrenic patients [29] or psychiatric outpatients [17], the French version of the BMQ appeared as a three-dimensional scale in SMI patients. It is interesting to note that this factor structure appeared stable whatever the psychiatric diagnosis of the included patients.

From a methodological perspective, most previous validation studies of the BMQ revealing the same factor structure as the original scale used the Kaiser criterion (eigenvalues > 1 rule) to identify the number of factors as the primary criterion [30–32]. Our findings also showed a two-factor solution for the BMQ-General based on the Kaiser criterion, but only one factor solution according to the scree plot and parallel analysis criteria. In subgroups of patients, on the basis of the Kaiser criterion, the PCA revealed between three and four factors for the BMQ-specific and between one and three factors for the BMQ-General (Table 2). A recent systematic review assessed which factor analysis determined the correct number of latent dimensions when applied to ordered-categorical survey items (specifically for five-category Likert items) [24]. Strong consensus recommended avoiding use of the Kaiser criterion because it can overestimate the number of factors, not considering the number of items tested and the sample size. Parallel analysis appeared to be the best choice of eigenvalue-based criteria for assessing dimensionality. In line with these recommendations, and according to our findings, we can formulate the hypothesis that previous validation studies could have overestimated the number of factors for the BMQ-General due to the limitations of the Kaiser criterion.

The second explanation could be related to the specificity of the SMI population concerning their general beliefs about medications in comparison with other chronic illness populations. As Beck et al. pointed out, the monofactorial solution for the BMQ-General suggests that SMI patients do not differ between harm and overuse of medication in general [29]. In line with these results, a previous study of SMI patients showed that neither BMQ-General subscale appears to measure independent dimensions, because Harm and Overuse subscales were strongly inter-correlated [18].

The PCA revealed the same items for each factor as the original scale in the total sample and in patients with bipolar disorder. In the schizophrenia subgroup, the Specific item 5 (“I sometimes worry about the long-term effects of my medicines”) did not reach a loading greater than 0.40 for the either factor, even if the higher loading was obtained for the Concerns factor than in the original scale. Unexpected and well-nigh incomprehensible was the higher loading of Specific item 9 (“I sometimes worry about becoming too dependent on my medicines”) on the Necessity factor in patients with major depressive disorder. We can hypothesise that there is a potential cultural effect on this loading. In a recent large epidemiological study in France, major depressive episodes remained associated with a high co-prescription rate of long-term treatment with benzodiazepines in comparison to other Western countries [33], treatment that is well-known in the general population to be associated with dependence. In this context, we can make the hypothesis that French patients may consider that the necessary treatment for major depressive disorder is associated with a risk of dependence. Another explanation could be due to the lack of representativeness of the sample because patients were only recruited in two French mental health hospital sites.

The means Necessity, Concerns and General scores of our whole sample and our three subgroups were similar to previous studies using BMQ with schizophrenic patients [11, 28], with mixed sample of patients with bipolar disorder and schizophrenia [18] or with psychiatric outpatients [17].

Both BMQ-Specific subscales and the BMQ-General scale showed satisfactory internal consistency, except for the BMQ-Specific Concerns in the subgroups of patients with schizophrenia and major depressive disorder. The significant correlations of the BMQ-Specific subscales and the BMQ-General scale with the DAI suggested the criterion validity of the BMQ to assess patients’ attitudes towards medications for all groups.

However, this study presents several limitations. The main limitation is the cross-sectional design, which cannot support causal relationships, along with the absence of data testing the test-retest reliability. The inclusion of SMI patients who consented to participate in the study represents a potential selection bias that can overestimate the percentage of patients with positive attitudes towards medication. The findings also need to be interpreted with caution due to the minimal sample size reached in each subgroup of patients [34] and the limited number of French hospital sites for inclusions. Finally, it will be interesting to use cross-validation procedures in future work to test models.

## Conclusion

Our findings highlight the interest to explore the factor structure and the psychometric properties of a scale in specific populations, because the theoretical equivalence between our population sample and patients with diabetes or HIV populations from the first French validation study of the BMQ was partially supported.

This questionnaire represents a useful tool to facilitate the identification of non-adherence and to implement strategies to improve negative beliefs in clinical practice. Future studies using the French version of the BMQ in patients with schizophrenia, bipolar disorder or major depressive disorder will be needed to confirm our findings.
